# The association of chronic, enhanced immunosuppression with outcomes of diabetic foot infections

**DOI:** 10.1002/edm2.298

**Published:** 2021-10-05

**Authors:** Ilker Uçkay, Madlaina Schöni, Martin C. Berli, Fabian Niggli, Emil Noschajew, Benjamin A. Lipsky, Felix W. A. Waibel

**Affiliations:** ^1^ Infectiology Balgrist University Hospital University of Zurich Zurich Switzerland; ^2^ Diabetic Foot Unit Department of Orthopedic Surgery Balgrist University Hospital University of Zurich Zurich Switzerland; ^3^ Internal Medicine Balgrist University Hospital University of Zurich Zurich Switzerland; ^4^ Department of Medicine University of Washington Seattle WA USA

**Keywords:** clinical failures, diabetic foot infection, enhanced immunosuppression, epidemiology, risk factors

## Abstract

We investigated if a chronic, enhanced immunosuppressed condition, beyond the immunodeficiency related to diabetes, is associated with clinical failures after combined surgical and medical treatment for diabetic foot infection (DFI). This is a case‐control cohort study in a tertiary centre for diabetic foot problems, using case‐mix adjustments with multivariate Cox regression models. Among 1013 DFI episodes in 586 patients (median age 67 years; 882 with osteomyelitis), we identified a chronic, enhanced immune‐suppression condition in 388 (38%) cases: dialysis (85), solid organ transplantation (25), immune‐suppressive medication (70), cirrhosis (9), cancer chemotherapy (15) and alcohol abuse (243). Overall, 255 treatment episodes failed (25%). By multivariate analysis, the presence (as compared with absence) of chronic, enhanced immune‐suppression was associated with a higher rate of clinical failures in DFI cases (hazard ratio 1.5, 95% confidence interval 1.1–2.0). We conclude that a chronic, enhanced immune‐suppressed state might be an independent risk factor for treatment failure in DFI. Validation of this hypothesis could be useful information for both affected patients and their treating clinicians.

## Abstract 2

A chronic, enhanced immune‐suppressed state (beyond the diabetes mellitus) is independent risk factor for treatment failure in diabetic foot infection.

## 1 INTRODUCTION

In the past few decades, the number of scientific publications concerning diabetic foot infections (DFI) has increased exponentially.^1^, ^2^ These have uncovered several clinical variables that are associated with failure of treatment for DFI, especially severe peripheral arterial disease, presence of osteomyelitis, insufficient surgical debridement and inadequate weight off‐loading.^1^, ^2^ It is known that diabetes mellitus is associated with defects in immune responses of both innate (including dysfunction of neutrophils and macrophages) and adaptive (including T cells) types.^3^ Patients with diabetes can also suffer from a chronic, ‘enhanced’ immunosuppressed state when they also are afflicted with various serious co‐morbidities. To our knowledge, there are no data assessing the influence of this type of immune suppression on the outcome of treatment for DFI. In our tertiary referral medical centre, we have been seeing an increasing number of patients with DFI who are afflicted with complex co‐morbidities and immune‐compromised states. Thus, we investigated the potential effects of these complications on the likelihood of successful treatment for their DFI.

## 2 METHODS

We performed a single‐centre, case‐control study targeting the primary outcome of ‘clinical failure’ after treatment for DFI in adult patients. We identified potential cases to include in our study using a DFI registry that listed all episodes we saw since the year 2000. We defined DFI and related osteomyelitis (DFO) based on the criteria in the diabetic foot infection guidelines published by the International Working Group on the Diabetic Foot (IWGDF) criteria.^1^ We defined the chronic, enhanced immunosuppressed state as being present in patients who required renal dialysis, had undergone organ transplantation requiring medical immune‐suppression, had advanced cirrhosis (CHILD B and C), were undergoing current chemotherapy for cancer, suffered from alcohol abuse (according to the patient, his/her family or the general practitioner) or were being treated with immune‐suppressing drugs. We did not include patients in this category who had an acute or transient cause of immunosuppression, such as polytrauma. We defined ‘clinical failure’ of DFI treatment as either a persistent, recurrent or new infectious problem at the original site. We defined ‘microbiological recurrence’ as a clinically persistent or recurrent DFI at the same localization from which the same pathogen(s) were isolated as before treatment was begun. This investigation is one of a retrospective group of studies (DF‐MANAG) approved by our medical centre's Ethical Committee (BASEC 2019–01994).

### 2.1 Statistical analyses

Our primary outcome of interest was whether clinical failure was related to the presence of chronic, enhanced immunosuppression. Our secondary outcome of interest was the risk, and any associations related to, microbiological recurrence. We compared groups with and without chronic, enhanced immunosuppression using the Pearson chi‐square or the Wilcoxon rank‐sum test. To adjust for the substantial case‐mix, we performed multivariate Cox regression analyses with both outcomes. We checked for collinearity and effect modification by interaction terms. Since ‘alcohol abuse’ was the only subjective parameter composing the variable ‘chronic, enhanced immunosuppression’, we ran all analyses twice—both with and without alcohol abuse embedded in the definition of immunosuppression.

We used STATA^™^ software (Version 15; College Station, TX).

## 3 RESULTS

### 3.1 Study population and infections

Among 1,013 DFI episodes in 586 patients (794 males; median age 67 years; 882 with DFO; 54 with concomitant Charcot neuroarthropathy), we identified a chronic, enhanced immunosuppression condition in 388 (38%) of the cases: renal transplantation (*n* = 20); other solid organ transplantation (5); immunosuppressive medication (70; 15 different drugs); chemotherapy for cancer (15); renal dialysis (85); advanced cirrhosis (9); and alcohol abuse (243). The patients’ overall median duration of diabetes history at our first consultation with them was 19 years.

### 3.2 Treatment and outcomes

The median number of surgical debridements per DFI episode was one; 572 (56%) of the patients underwent angioplasty of the affected limb. Results of cultures yielded 96 different bacterial constellations, which were treated with 46 different antimicrobial agents (often in combination). Overall, treatment failure occurred in 255 episodes (25%), while in the remaining 758 (75%), there was long‐term remission of the DFI episode. The median active follow‐up for this cohort of patients was 7.7 years. Among the 255 clinical failures, 47 (5%) had a microbiological recurrence (representing 18% of all failures).

By crude group comparisons, we found no difference in clinical failures in the patients with or without chronic, enhanced immunosuppression (107/388 [28%] vs. 148/625 [24%]; *p*=0.17; Table 1). Similarly, there was no difference in the rate of microbiological recurrence between those with or without chronic, enhanced immunosuppression (20/388 [5%] vs. 27/625 [4%]; *p*=0.54). However, after case‐mix adjustment with multivariate Cox regression (Table 2), we found that chronic, enhanced immunosuppression was significantly associated with clinical failure in all analyses (hazard ratio 1.5, 95% confidence interval 1.1–2.0). We re‐confirmed this finding even after omitting alcohol abuse from the definition of chronic, enhanced immunosuppression (HR 1.5, 95%CI 1.1–2.1). In contrast, chronic, enhanced immunosuppression was not associated with microbiological recurrence in the multivariate analysis (HR 1.2; 95%CI 0.6–2.8; Tables 1 and 2). All of the receiver‐operating‐curve (ROC) values were >0.72, suggesting good accuracy of our multivariate models.

**TABLE 1:** Characteristics and outcomes of 1,013 patients with a diabetic foot infection

*n* = 1013	Clinical failure	Remission without failure	*p*‐value^b^
	*n* = 255 (25%)	*n* = 758 (75%)	
Male sex	203 (80%)	591 (78%)	.58
Median age (years)	65	68	.09
Enhanced immune‐suppression (with alcohol abuse)^a^	107 (42%)	281 (37%)	.17
Enhanced immune‐suppression (without alcohol abuse)	97 (38%)	251 (33%)	.15
Diabetic foot osteomyelitis	213 (84%)	669 (88%)	.06
Duration of diagnosed diabetes mellitus (median)	20 years	18 years	. ***03***
Number of surgical debridement (median)	1	1	. ***01***
Duration of antibiotic therapy (median)	30 days	20 days	. ***01***
Need for lower extremity revascularisation	164 (64%)	408 (54%)	. ***01***

^a^ Enhanced immunosuppression =solid organ transplants, cirrhosis CHILD B and C, renal dialysis, chemotherapy for cancer and steroids,

^b^ Pearson chi‐square test or Wilcoxon rank‐sum tests. Significant results (*p*<0.05) are indicated ***in bold and italic***.

**TABLE 2:** Univariate and multivariate associations *(Cox regression analyses with results expressed as hazard ratios with 95% confidence intervals)* with the outcome ‘clinical failure’ and ‘microbiological recurrence’ in patients with or without alcohol abuse included as immunosuppression (IS)

Clinical failures *Alcohol abuse is part of IS*	Univariate	Multivariate	Clinical failures *Alcohol abuse not IS*	*Multivariate*	Microbiological recurrences *Multivariate results with alcohol being an IS*
Age	1.0, 1.0–1.0	‐	‐	‐	1.0, 1.0–1.0
Enhanced immune‐suppression	***1.4, 1.1–1.7***	***1.5, 1.1–2.0***	‐	***1.5, 1.1–2.1***	1.2, 0.6–2.8
Diabetic foot osteomyelitis	1.1, 0.8–1.5	1.2, 0.8–1.8	‐	1.2, 0.8–1.8	‐
Diabetes mellitus type I	1.1, 0.8–1.5	1.1, 0.7–1.7	‐	1.0, 0.7–1.6	‐
Duration of diagnosed diabetes	1.0, 1.0–1.0	‐	^−−^	‐	1.0, 1.0–1.0
Peripheral arterial disease	1.1, 0.8–1.5	1.0, 0.7–1.5	‐	1.0, 0.7–1.6	‐
Need for revascularisation	1.2, 0.9–1.5	1.1, 0.8–1.5	‐	1.1, 0.8–1.5	0.6, 0.3–1.3
Duration of antibiotic therapy	1.0, 1.0–1.0	1.0, 1.0–1.0	‐	0.5, 0.3–1.0	1.0, 1.0–1.0
‐ Intravenous therapy	1.0, 1.0–1.0	‐	‐	1.0, 1.0–1.0	1.0, 1.0–1.0

Note

Statistically significant results are displayed in bold and italic; ‘‐‘, not included in the model.

Of note, we found that in this study population almost all clinical failures were surgically revised (253/255 [99%]), of which 149 (58%) consisted of a major amputation. Furthermore, we found that 290 of our 1013 DFI cases (29%) died during the prolonged study follow‐up, at a median of 3.5 years following treatment for the index infection.

## 4 DISCUSSION

We found that in our large population of patients with DFI that the presence of a chronic, enhanced immunosuppressing condition was significantly associated with clinical treatment failure (25% of cases). The incidence of microbiological recurrences was, however, low (5% of all DFI cases or 18% of the failures) and was not statistically associated with chronic, enhanced immunosuppression. Hence, we associate an enhanced immunosuppression rather with wound breakdowns than with insufficient anti‐infectious effects of DFI treatment.

It is perhaps not surprising that the outcome of treatment for DFI would be worse in patients with immunosuppressing conditions, as this is in line with finding in many other infections. But the literature on this topic is very limited, and to our knowledge, this is the first study to address the question. We were unable to find any previous published investigations on the role of immunosuppressive conditions beyond those specifically associated with diabetes in DFI patients, except in selected situations, such as those undergoing renal dialysis^4^, ^5^ or having a renal transplant,^6^ or addressing risk factors for diabetic foot ulcers^5^, ^7^ or mortality.^4^ Zou and Wukich reported that diabetic patients with solid transplant have no increased risk for nosocomial DFI after foot surgery (odds ratio 0.5, 95%CI 0.1–3.1).^6^

We examined definitions of chronic, enhanced immunosuppression both including and excluding alcohol abuse,^5^, ^7^ and the findings were the same. We undertook this assessment because of the high prevalence of elevated alcohol consumption among our DFI population (243/1,013; 24%). In a recent Chinese survey, the prevalence of ‘current’ alcohol consumption among patients with diabetic foot ulcers was 35.3%.^7^ Among the participants in the Eurodiale trial targeting diabetic foot ulcers, prevalence of alcohol consumption was 45%.^5^ Some investigators have suggested alcohol abuse might increase the risk of diabetic foot ulcers.^8^ This may occur by several different pathways, including the possibility that alcohol impairs the proliferative phase of wound healing.^9^

The main strengths of our brief report are the large database (over 1000 DFI patients) with a long follow‐up (almost 8 years) in a specialized, academic diabetic foot unit. The main limitations are the varied case‐mix of our DFI population and the lack of proof of a causal relationship between the significant association of chronic, enhanced immune suppression and clinical treatment failure. Based on available information, we devised the term chronic, enhanced immunosuppression and selected the various medical conditions used in its definition. We are aware, of course, that DFIs are a highly variable group of entities with different pathogens, levels of tissue involvement, concomitant co‐morbidities, geographical settings, and vascular and surgical problems.^10^ In addition, we understand that there is even greater heterogeneity in immune‐comprising conditions. In view of the varied composition of our study population, we were not able to perform separate and stratified analyses for each potential immunodeficiency or DFI episode, and had to rely on doing multivariate analyses. Our results only allow us to identify chronic, enhanced immunosuppression as only as a risk association. It could reflect more the presence of multiple co‐morbidities and the affected patients’ frailty, instead of being causal for treatment failure *per se*.

In conclusion, we suggest that chronic, enhanced immunosuppression, beyond that related to diabetes alone, might be a risk factor for treatment failure in patients with DFI. If this hypothesis is validated by further studies, it would be useful information for both the increasing numbers of affected patients and their treating clinicians.

## CONFLICT OF INTERESTS

None of the authors have any financial or other conflicts of interest with this work.

## AUTHOR CONTRIBUTION

**Ilker Uçkay:** Conceptualization (lead); Data curation (equal); Formal analysis (lead); Investigation (lead); Methodology (lead); Project administration (equal); Validation (lead); Writing‐original draft (lead); Writing‐review & editing (lead). **Madlaina Schöni:** Conceptualization (equal); Formal analysis (equal); Investigation (lead); Methodology (supporting); Resources (supporting); Validation (equal); Visualization (equal); Writing‐original draft (supporting); Writing‐review & editing (supporting). **Martin C. Berli:** Data curation (supporting); Project administration (supporting); Resources (supporting); Supervision (supporting); Visualization (lead); Writing‐review & editing (supporting). **Fabian Niggli:** Data curation (lead); Investigation (supporting); Project administration (supporting); Resources (lead). **Emil Noschajew:** Conceptualization (supporting); Data curation (supporting); Formal analysis (supporting); Investigation (supporting); Resources (supporting); Visualization (supporting). **Benjamin A Lipsky:** Conceptualization (supporting); Methodology (supporting); Supervision (equal); Validation (supporting); Writing‐original draft (equal); Writing‐review & editing (equal). **Felix Waibel:** Conceptualization (supporting); Data curation (lead); Investigation (equal); Methodology (equal); Project administration (lead); Resources (equal); Validation (supporting); Writing‐review & editing (supporting).

## Data Availability

The corresponding author can provide anonymized key data upon reasonable scientific request.
